# Pharmacokinetics, optimal dosing, and safety of linezolid in children with multidrug-resistant tuberculosis: Combined data from two prospective observational studies

**DOI:** 10.1371/journal.pmed.1002789

**Published:** 2019-04-30

**Authors:** Anthony J. Garcia-Prats, H. Simon Schaaf, Heather R. Draper, Maria Garcia-Cremades, Jana Winckler, Lubbe Wiesner, Anneke C. Hesseling, Rada M. Savic

**Affiliations:** 1 Desmond Tutu TB Centre, Department of Paediatrics and Child Health, Faculty of Medicine and Health Sciences, Stellenbosch University, Tygerberg, South Africa; 2 Department of Bioengineering and Therapeutic Sciences, University of California, San Francisco, San Francisco, California, United States of America; 3 Division of Clinical Pharmacology, Department of Medicine, University of Cape Town, Cape Town, South Africa; FIND, SWITZERLAND

## Abstract

**Background:**

Linezolid is increasingly important for multidrug-resistant tuberculosis (MDR-TB) treatment. However, among children with MDR-TB, there are no linezolid pharmacokinetic data, and its adverse effects have not yet been prospectively described. We characterised the pharmacokinetics, safety, and optimal dose of linezolid in children treated for MDR-TB.

**Methods and findings:**

Children routinely treated for MDR-TB in 2 observational studies (2011–2015, 2016–2018) conducted at a single site in Cape Town, South Africa, underwent intensive pharmacokinetic sampling after either a single dose or multiple doses of linezolid (at steady state). Linezolid pharmacokinetic parameters, and their relationships with covariates of interest, were described using nonlinear mixed-effects modelling. Children receiving long-term linezolid as a component of their routine treatment had regular clinical and laboratory monitoring. Adverse events were assessed for severity and attribution to linezolid. The final population pharmacokinetic model was used to derive optimal weight-banded doses resulting in exposures in children approximating those in adults receiving once-daily linezolid 600 mg. Forty-eight children were included (mean age 5.9 years; range 0.6 to 15.3); 31 received a single dose of linezolid, and 17 received multiple doses. The final pharmacokinetic model consisted of a one-compartment model characterised by clearance (CL) and volume (V) parameters that included allometric scaling to account for weight; no other evaluated covariates contributed to the model. Linezolid exposures in this population were higher compared to exposures in adults who had received a 600 mg once-daily dose. Consequently simulated, weight-banded once-daily optimal doses for children were lower than those currently used for most weight bands. Ten of 17 children who were followed long term had a linezolid-related adverse event, including 5 with a grade 3 or 4 event, all anaemia. Adverse events resulted in linezolid dose reductions in 4, temporary interruptions in 5, and permanent discontinuation in 4 children. Limitations of the study include the lack of very young children (none below 6 months of age), the limited number who were HIV infected, and the modest number of children contributing to long-term safety data.

**Conclusions:**

Linezolid-related adverse effects were frequent and occasionally severe. Careful linezolid safety monitoring is required. Compared to doses currently used in children in many settings for MDR-TB treatment, lower doses may approximate current adult target exposures, might result in fewer adverse events, and should therefore be evaluated.

## Abstract 2

Using data from two cohorts, Anthony Garcia-Prats and colleagues model the appropriate dosing necessary to achieve target concentrations for linezolid to treat children with multidrug-resistant tuberculosis.

## Author summary

**Why was this study done?:**

- Linezolid is an antibiotic that has recently been repurposed for the treatment of multidrug-resistant tuberculosis (MDR-TB).
- Based on emerging data from clinical trials, it is now recommended for inclusion as a priority in treatment regimens for adults and children.
- There is a lack of evidence-based dosing guidelines for linezolid in children for MDR-TB.
- Linezolid has frequent adverse effects that are associated with higher doses and longer durations of treatment, but this has not been well described in children treated for MDR-TB.

**What did the researchers do and find?:**

- We assessed linezolid pharmacokinetics (concentrations) and safety among children routinely treated for MDR-TB from 2 observational studies.
- We modelled this pharmacokinetic data and simulated the doses in children that would be needed to achieve current targets.
- Doses supported by our data for some weight bands were lower than currently in use, which may reduce the risk of adverse effects.
- Linezolid-related adverse effects were frequent—with low haemoglobin being the most common—and occasionally severe (grade 3 or 4).

**What do these findings mean?:**

- Revised linezolid doses supported by this study now provide an evidence base for international dosing recommendations.
- All linezolid-treated children with MDR-TB should have careful safety monitoring to include at least regular haemoglobin monitoring.
- Larger studies, evaluating the revised dosing, are needed to better characterise linezolid safety and to assess the risk of more rare linezolid-related events, especially given the small number of children in this study.

## Introduction

Multidrug-resistant (MDR) tuberculosis (TB) continues to threaten global TB control, with an estimated 460,000 incident cases worldwide in 2017 [1]. Treatment options remain limited. Linezolid, an oxazolidinone antibiotic that binds to the 50S ribosomal subunit inhibiting protein synthesis [2], is increasingly being used for MDR-TB treatment. In routine use in adults with MDR-TB, linezolid has been associated with good outcomes, with 68% and 82% of patients successfully treated in 2 systematic reviews [3, 4]. In a recent systematic review and individual patient data meta-analysis of 12,030 patients with MDR-TB, treatment with linezolid was significantly associated with treatment success compared to failure or relapse, and also with reduced mortality [5]. The World Health Organisation (WHO) released updated guidance of MDR-TB treatment in 2018, which now classifies linezolid as a Group A drug, meaning that it is a priority to include in individually constructed MDR-TB regimens for adults and children [6].

Interest in linezolid has grown further based on the preliminary results of the Nix-TB trial (NCT02333799), a single-arm open-label phase III study that evaluated very difficult to treat adults with extensively drug-resistant (XDR)-TB or MDR-TB treatment intolerance, or failure, with a three-drug regimen of bedaquiline, pretomanid, and linezolid (1,200 mg given once daily) for 6 months [7]. In an interim analysis of the first 75 patients, 6 died, and all the remaining patients completed the 6 months of treatment; 66 (88%) had durable cure 6 months later, and there were only 2 relapses [7]. Linezolid is also a component of multiple other novel, shortened MDR-TB treatment regimens currently under evaluation in adults [8].

Used in short courses (<28 days), linezolid is safe and well tolerated, but with the longer treatment durations being used for MDR-TB treatment (typically 6 months or longer), it is associated with frequent serious, dose- and duration-dependent adverse effects, including anaemia, neutropaenia, thrombocytopaenia, peripheral neuropathy, and more rarely, optic neuropathy, lactic acidosis, pancreatitis, and rhabdomyolysis [9]. In the systematic reviews of linezolid-treated adults with MDR-TB, linezolid-related adverse effects were reported in 61% and 59% of patients, respectively [3, 4], with 69% of these requiring linezolid dose adjustment or discontinuation in one review [3]. A linezolid dose of >600 mg daily was associated with an increased risk of adverse effects in these reviews [3, 4]. In early interim analysis of the Nix-TB study, 27% of participants experienced a serious adverse event, and 71% of participants had at least one linezolid-related treatment interruption, due mostly to myelosuppression or peripheral neuropathy [10].

There is a substantial burden of MDR-TB in children, with an estimated 25,000 to 32,000 incident cases globally each year [11, 12], many of whom could benefit from linezolid treatment. The evidence base for linezolid use in children with MDR-TB is currently limited. A 2014 review of the literature identified only case reports and small case series that described 18 linezolid-treated children with MDR-TB [9]. As in adults, treatment outcomes were good, with 15 of 18 children (83%) successfully treated, but 9 (50%) experienced a linezolid-related adverse effect, 5 (28%) required dose adjustment, and 2 (11%) discontinued linezolid [9]. These limited early data were retrospective and had variable reporting of key information. High-quality prospective data are needed to better characterise the incidence, severity, and timing of adverse events among children with MDR-TB receiving long-term linezolid (>28 days of treatment).

The optimal dose of linezolid for treatment of adults with MDR-TB is not yet defined. However, 600 mg once daily is currently the most frequently used routine dose in adults. The dose of linezolid in children needed to approximate target exposures in adults with MDR-TB receiving a 600 mg daily dose has not yet been characterised. Linezolid is well absorbed after oral administration, with bioavailability of nearly 100% [2, 13]. Linezolid has a complex metabolism, with the primary metabolite formed through a nonenzymatic mechanism and the major pathway of elimination urinary excretion [13]. Linezolid has good penetration into tissues, including cerebrospinal fluid (CSF), where its exposure is 70% to 98% of plasma exposure [13–15]. Studies of linezolid pharmacokinetics in adults to date have shown substantial variability between patients, patient populations, and studies, with limited data in patients with TB [16–18].

Linezolid pharmacokinetics has been studied in children with gram-positive infections [19]; however, there are no data in children treated for TB. This gap is important, because dosing guidance should ideally be based on data from the target population, particularly as linezolid pharmacokinetics is known to vary considerably across disease states [18]. Understanding the optimal dose of linezolid for MDR-TB treatment in children is critical, because too high a dose may increase the risk of serious adverse effects, while underdosing may reduce the efficacy of this drug and may potentially lead to the development of linezolid resistance [20, 21]. Practical, weight-banded paediatric dosing guidance for linezolid based on its pharmacokinetics in children with TB is urgently needed as its use is likely to increase substantially based on the revised 2018 WHO guidance.

The objective of this study was to characterise the pharmacokinetics and safety of linezolid in children routinely treated for MDR-TB and to estimate optimal weight-banded doses that achieve adult target exposures for MDR-TB treatment.

## Methods

### Study design, setting, and population

The data described here are combined from 2 prospective observational pharmacokinetic studies in Cape Town, South Africa, which used standard clinical study measures, identical drug formulations, and standard sample collection and laboratory methods. The first observational study (MDRPK1) enrolled HIV-infected and -uninfected children 0 to <15 years of age (n = 173) routinely treated with second-line anti-TB drugs for probable or confirmed drug-resistant TB, who were followed until the end of MDR-TB treatment, from 2011 to 2015. All children from this cohort routinely treated with linezolid were included in the current analysis. The second study (MDRPK2) was a direct follow-up observational study to MDRPK1 and enrolled HIV-infected and -uninfected children 0 to <18 years of age (n = 64) routinely treated for MDR-TB with levofloxacin, moxifloxacin, or linezolid, in the same setting, from 2016. Follow-up for MDRPK2 is ongoing. In MDRPK2, children not prescribed linezolid as a component of their routine MDR-TB care received a single dose of linezolid on the day of pharmacokinetic sampling and therefore contributed data to pharmacokinetic analyses but not to long-term safety data. The total sample size for MDRPK2 was estimated from clinical trial simulations with existing models to ensure sufficient precision primarily of the pharmacokinetic parameter estimates (relative standard error [RSE] of parameter estimates of <10%) (see S1 Text). The minimum overall sample size identified was 80 evaluable participants, with 100 participants being the maximum targeted sample size to allow for attrition. The present report is based on a planned interim analysis after enrolment of 50 participants; 9 patients from MDRPK1 were added to supplement participants from MDRPK2. The timing of this analysis (which included just under the planned 50 participants) was also influenced by the lack of a strong evidence base for linezolid dosing for TB in children and the urgent need for data, as well as the timing of the revision of WHO MDR-TB treatment guidelines and the need to inform that process in the absence of other paediatric data.

Children with MDR-TB were treated individually according to national and international guidelines—with a minimum of 4 confirmed- or likely-effective drugs, usually with the addition of pyrazinamide and ethambutol—generally for 12 to 18 months’ duration [22–24]. Due to its cost and adverse effects, at the time of the study, linezolid was reserved for children with MDR-TB with (1) probable or confirmed additional fluoroquinolone resistance, including those with XDR-TB; (2) MDR-TB meningitis; (3) or documented intolerance to other second-line anti-TB medications. Management of linezolid-associated adverse effects was individualised depending on the event type and severity, the child’s TB disease severity, and the availability of other treatment options. For common, nonsevere events such as low-grade (grades 1 or 2) anaemia, linezolid would typically be temporarily interrupted and then restarted at half the dose. For more severe events (grade 3 or 4), linezolid may have been permanently discontinued, depending on other treatment options and the child’s clinical status at the time. These clinical decisions were individualised and were made at the discretion of the routine treating clinician.

### Linezolid dosing and pharmacokinetic sampling

At the time of the study, there was no dosing recommendation based on high-quality evidence for linezolid for TB treatment in children. The routinely prescribed dose of linezolid in the study setting, based on expert opinion, was 10 mg/kg/dose twice daily (20 mg/kg daily) for children <10 years of age and 10 mg/kg/dose once daily for children >10 years of age, up to a maximum total daily dose of 600 mg. This was based on an approximation of the dose required to achieve similar exposure as in adults receiving 600 mg once daily, based on existing pharmacokinetic data from children with non-TB infections [9]. Linezolid was available as 600 mg unscored tablets and as a 20 mg/mL suspension (Pfizer, Sandton, South Africa).

In the MDRPK1 study, an exact 10 mg/kg dose was prepared and administered along with other anti-TB medications in the regimen on the day of pharmacokinetic sampling. Samples were drawn just prior to—and then at 1, 2, 4, 8, and either 6 or 11 hours after—the anti-TB medications’ dose. In MDRPK2, a weight-banded dose (approximately 10 mg/kg) was administered on pharmacokinetic sampling days along with other routine anti-TB medications in the regimen. Samples were drawn just prior to, and at 1, 4, and 10 hours after, the observed dose. For both studies, medications were administered identically, on an empty stomach after an overnight fast, and were given either as whole tablets if the child was able to swallow, as a suspension if available, or as crushed tablets mixed in a small amount of water if the child was unable to swallow and the suspension was unavailable (due to occasional stockout), using identical formulations in both studies. On occasion, all anti-TB medications were administered via nasogastric tube on pharmacokinetic sampling days, only if the child refused to swallow medications orally (e.g., young children). One hour after the anti-TB medication dose, antiretroviral medications were administered in HIV-infected children, and a standard breakfast was offered.

### Laboratory assays

Pharmacological assays were performed at the University of Cape Town, Division of Clinical Pharmacology using a validated liquid chromatography tandem mass spectrometry (LC MS/MS) method. This LC MS/MS method involves a simple protein precipitation extraction using 20 μl plasma, followed by isocratic separation on a Poroshell 120EC-C18, 4.6 × 50 mm, 2.7 μm column. A deuterated internal standard, Linezolid-d3, is used to monitor the method across a calibration range of 0.100 μg/ml (LLOQ) to 30 μg/ml (ULOQ). The method performed well over the period of analysis, with an accuracy ranging from 96.6% to 98.7% and a precision estimate of less than 7.2% (% CV) over all 3 quality control (QC) sample concentrations (QC low at 0.25 μg/ml, QC medium at 12 μg/ml, and QC high at 24 μg/ml).

### Safety

All children had regular clinical and laboratory safety assessments, including a full blood count monthly for the first 6 months, then every 2 months or as clinically indicated until completion of treatment. All adverse events were recorded and were assessed for attribution to linezolid based on the judgment of the investigator or subinvestigator, who was not blinded to the presence or duration of linezolid treatment, and graded for severity. In MDRPK1, events were graded according to standard Division of AIDS (DAIDS) criteria (“DAIDS Table for Grading the Severity of Adult and Pediatric Adverse Events” version 1.0, December 2004, updated August 2009) [25]. In MDRPK2, events were graded according to the updated DAIDS Table for Grading the Severity of Adult and Pediatric Adverse Events (version 2.0, November 2014 [26]; corrected version 2.1, July 2017 [27]). There were no differences between these versions for most of the adverse events that are relevant to linezolid, including low platelets, low white blood cell count, and peripheral neuropathy; the grading for these events is shown in S1 Table. There were slight differences in the way low haemoglobins were graded, which are summarised separately in S2 Table. The minor differences in low haemoglobin grading are unlikely to have meaningfully changed the results or the study’s overall conclusions.

### Statistical analysis

The full MDRPK2 study protocol Statistical Considerations section is included as S1 Text. The analyses described here and in the following section are consistent with the general statistical approach in the protocol. Baseline characteristics were presented with descriptive statistics. Weight-for-age (WAZ) and height-for-age (HAZ) z-scores were calculated using British reference values, as WHO references only include children <10 years of age [28]. Only children receiving linezolid as a component of their routine drug-resistant TB treatment regimen were included in safety analyses (i.e., not children only receiving single-dose linezolid). Adverse events at least possibly related to linezolid were presented by grade, and the rate of events per person-time of observation were calculated. The development of a pharmacokinetic-pharmacodynamic model to assess the relationship between linezolid exposures and adverse events was planned after the study was fully enrolled and completed. At the time of the current analysis, this was explored using the analyses described below. The median (interquartile range [IQR]) linezolid area under the concentration time curve from 0 to 24 hours at steady state (AUC_0–24ss_) was reported and compared by the presence of linezolid-related adverse effects using the Wilcoxon rank sum test. Linezolid exposure was further defined using AUC_0–24ss_ and split into 2 groups using the median AUC_0–24ss_, 107 mg/L × h. Survival curves for time to any linezolid-related adverse effect and time to grade 3 or 4 linezolid-related adverse effects were displayed using Kaplan-Meier plots. Patients were censored at the time of the relevant linezolid-related event or when linezolid was discontinued. The log-rank test was used to assess whether linezolid exposure was associated with the time to linezolid-related grade 3/4 or any-grade adverse events. If the proportional hazards assumption was violated, log-rank test was reported along with a comparison of the restricted mean survival times (RMSTs) [29, 30]. During the peer review process, these analyses were repeated to assess associations between the minimum linezolid concentrations (C_mins_) and linezolid-related adverse effects. A linezolid C_min_ of 2 mg/L, suggested as a threshold value for risk of adverse events [31], was used to separate groups in survival curves.

### Population pharmacokinetic model and dosing simulations

Pharmacokinetic data were characterised based on a population nonlinear mixed-effects modelling approach using the software NONMEM 7.41 (ICON Development Solutions, Ellicott City, Maryland). The method of estimation used was the first order conditional estimation (FOCE) method with the option ‘interaction’. Between-subject variability (BSV) was modelled exponentially, and the residual error was described using a combination of an additive and proportional error model. Inter-occasion and inter-cohort variability were also evaluated to deal with the differences between both studies and pharmacokinetic occasions. Model building was performed in 2 stages, with the structural model developed first and the covariate analysis done subsequently. The main covariates evaluated in the analysis were weight, height, age, sex, race, HIV status, linezolid administration method (oral versus nasogastric tube), formulation (whole versus crushed versus suspension), linezolid given as single dose versus at steady state, and interactions with concomitant drugs. Covariate identification was done using the stepwise covariate modelling (SCM) through the PsN software (version 4.6.0). This method consists of the stepwise testing of linear and nonlinear covariate-parameter relationships with forward inclusion and backward exclusion approaches with significance levels of 0.05 and 0.01, respectively. Given the modest sample size and the large number of covariates, the analysis was also done with a forward inclusion level of 0.2 and backward exclusion of 0.05. The final inclusion of the identified significant covariates was done taking into account scientific plausibility, statistical significance, and clinical relevance. Both stages of model building were evaluated by the likelihood ratio test, goodness of fit plots, and visual predictive checks (VPCs). R software (version 3.5.1) was used to make the graphical representation, using the xpose package (version 4.6.1) (reference: https://CRAN.R-project.org/package=xpose) for data set checkout and graphical evaluation of the results.

The final model was used to simulate different linezolid doses depending on children’s weights. Model-identified optimal doses were derived based on the following assumptions with respect to formulation availability: (a) independent of formulation, e.g., exact optimal doses, (b) optimal doses with 20 mg/mL suspension, and (c) optimal doses with 600 mg tablet in one-fourth tablet increments. The target linezolid exposure used for dosing simulations was the AUC_0–24ss_ in adults with TB after a 600 mg once-daily dose (110 mg/L^.^h). This target is based on data on linezolid pharmacokinetics from a completed phase II dose-ranging trial in adults with drug-susceptible TB (LINCL-001, NCT02279875, *N* = 99) and an ongoing phase III trial in adults with MDR-TB, (NiX-TB, NCT02333799, *N* = 88) (S4 Table). Two hundred simulations of the pharmacokinetic profile of a paediatric population with weights ranging from 5 to 56 kg, receiving the calculated weight-banded dose regimen, were performed in NONMEM (Icon Development Solutions, Ellicot City, MD), and their AUCs were reported.

This study is reported as per the Strengthening the Reporting of Observational Studies in Epidemiology (STROBE) guideline (S1 STROBE Checklist).

### Ethical considerations

Written informed consent was provided by the parent(s) or legal guardians, and written informed assent was given by participants ≥7 years of age. Ethical approval for the study was provided by the Health Research Ethics Committee of Stellenbosch University (N11/03/059 for MDRPK1 and N15/02/012 for MDRPK2).

## Results

### Study population

Forty-eight children were included in the present analysis, 9 from MDRPK1 and 39 from MDRPK2. Baseline clinical and demographic characteristics by study (MDRPK1, MDRPK2) are shown in Table 1.

**Table 1:** Baseline demographic and clinical characteristics of children with MDR-TB receiving linezolid including data from 2 observational pharmacokinetic studies.

Characteristic	MDRPK1 Multiple dose* (*n* = 9)	MDRPK2 Multiple dose (*n* = 8)	MDRPK2 Single dose (*n* = 31)	Combined (*n* = 48)
**Median age in years (range)**	4.8 (0.6–13.8)	4.9 (1.8–15.3)	4.4 (1.1–14.6)	4.6 (0.6–15.3)
**Male gender (%)**	6 (66.7)	6 (75.0)	12 (38.7)	24 (50.0)
**Ethnicity (%)**				
**Black**	4 (44.4)	6 (75.0)	15 (48.4)	25 (52.1)
**Mixed race**	5 (55.6)	2 (25.0)	16 (51.6)	23 (47.9)
**HIV-infected (%)**	1 (11.1)	1 (12.5)	1 (3.2)	3 (6.3)
**WAZ < −2 (%)**	3 (33.3)	3 (37.5)	3 (9.7)	9 (18.8)
**Administration on day of PK sampling**				
**NGT (%)**	6 (66.7)	0 (0.0)	6 (19.4)	12 (25.0)
**Oral (%)**	3 (33.3)	8 (100.0)	25 (80.6)	36 (75.0)
**Formulation**				
**Whole tablet (%)**	1 (11.1)	3 (37.5)	1 (3.2)	5 (10.4)
**Crushed tablet (%)**	1 (11.1)	1 (12.5)	8 (25.8)	10 (20.8)
**Suspension (%)**	7 (77.8)	4 (50.0)	22 (71.0)	33 (68.8)

*None of the MDRPK1 patients had single dose.

The median age (range) of the children receiving each type of formulation are 13.0 years (7.2–15.3) for whole tablet (*n* = 5), 12.3 years (2.7–15.4) for crushed tablet (*n* = 12), and 2.6 years (0.6–9.6) for suspension (*n* = 37).

**Abbreviations:** MDR-TB, multidrug-resistant tuberculosis; NGT, nasogastric tube; PK, pharmacokinetic; WAZ, weight-for-age z-score.

### Pharmacokinetic model

**Fig 1:**
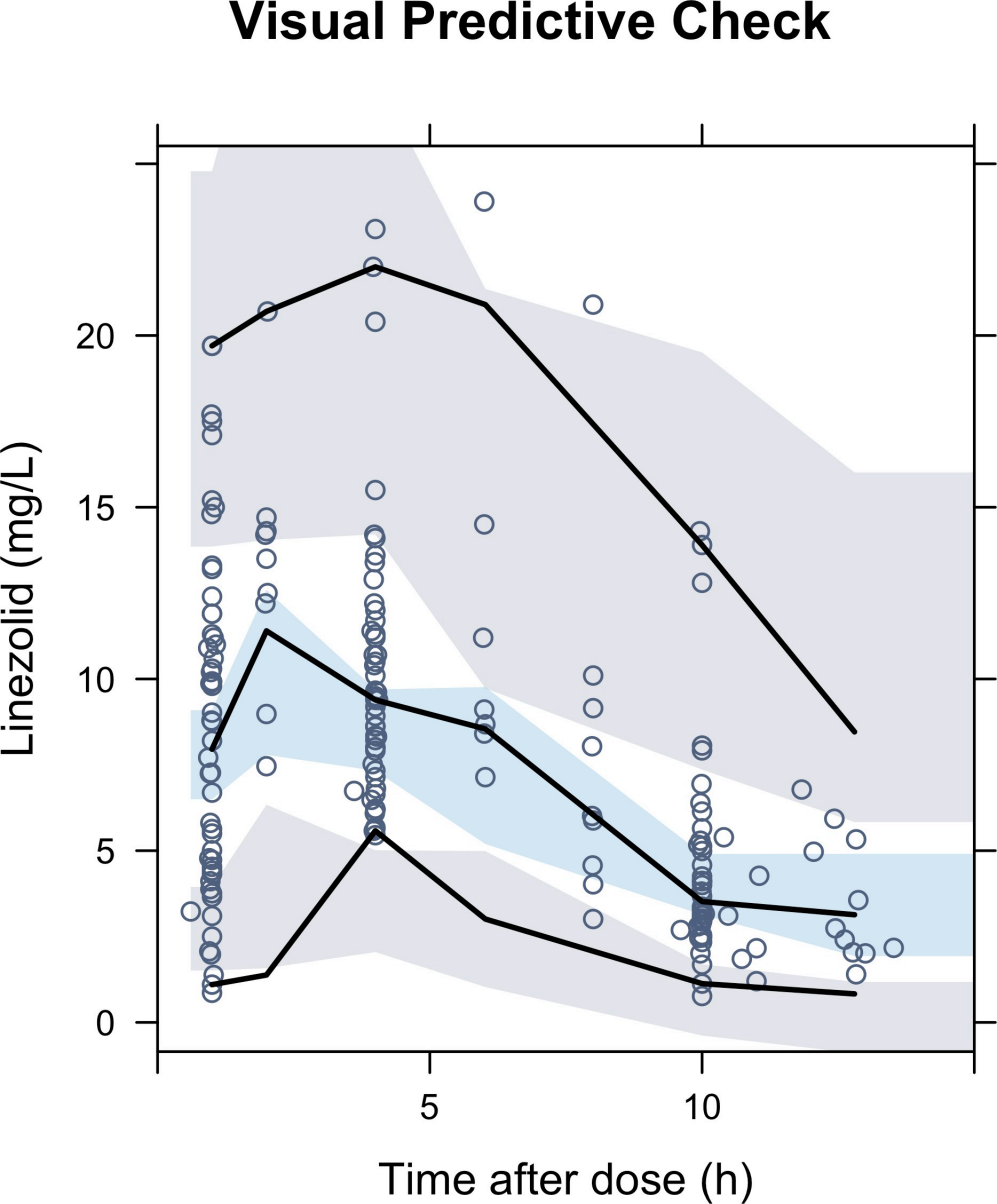
VPC corresponding to final linezolid pharmacokinetic model. Dots represent the observed linezolid concentrations; the solid lines correspond to the 5th, 50th, and 95th percentiles of these observations. Shaded areas are the model-predicted 95% confidence intervals for the median (blue), and 5th and 95th (grey) percentiles obtained from 500 simulated data sets. VPC, visual predictive check.

Of the 48 children included, 5 participants contributed data from more than 1 pharmacokinetic sampling occasion. Four patients from MDRPK2 contributed full profiles from 2 sampling occasions, and 1 patient from MDRPK1 contributed 1 full profile and 2 partial profiles. The pharmacokinetic profiles of these 5 children are displayed in S1 Fig, including the raw pharmacokinetic data, their individual prediction, and the dose and formulation received on each occasion. No concentrations were below the limit of quantitation. The final pharmacokinetic model consisted of a one-compartment disposition model characterised by clearance (CL) and volume (V) parameters. CL and V included allometric scaling, using the exponents 0.75 and 1, respectively, to account for changes in weight. The rate of absorption (K_a_) was constrained to be faster than the rate of elimination (CL/V) in order to avoid flip-flop kinetics during model estimation [32]. BSV was estimated on both CL and V. Inter-occasion and inter-cohort variability were assessed and were not included in the final model due to the lack of significance. This lack of significance may be explained by the small number of pharmacokinetic measurements available at second and third occasions (only 5 out of 48 children). Similarly, the lack of significance when using inter-cohort variability suggests that the populations between cohorts were similar and comparable. No other covariates tested—including age, HIV status, formulation (whole tablets versus crushed tablets versus suspension), WAZ, HAZ, body mass index, administration by nasogastric tube—significantly improved the model fit for any of the pharmacokinetic parameters after accounting for the effect of weight. S3 Table reports the coefficients and the 95% confidence intervals of several covariate models tested during the covariate analysis. Table 2 describes the final model parameters. The final model fit the observed data well, as shown in the VPC (Fig 1). Calculated AUC and maximum plasma concentrations from our study population are described in Table 3.

**Table 2:** Parameter values for modelled linezolid pharmacokinetics in children with MDR-TB (*n* = 48).

Parameter	Estimate	RSE (%)
K_a_ (h^−1^) θ_K_a_ + (CL/V)	θ_K_a_ = 0.77	25
CL/F (L/h) = θ_CL ×〖"(WT ÷ 70")〗^0.75	θ_CL = 4.73	8
V/F (L) = θ_V × (“WT ÷ 70”)	θ_V = 54.8	8
BSV_CL/F_ (%)	37	16
BSV_V/F_ (%)	32	23
Additive error (mg/L)	0.78	91
Proportional error (%)	25	30

**Abbreviations:** BSV, between-subject variability; CL, clearance; F, bioavailability; K_a_, absorption rate constant; RSE, relative standard error; V, volume of distribution; WT, weight in kg; θ, typical parameter.

**Table 3:** Calculated linezolid AUC_0–24ss_ and C_max_ from children with MDR-TB (*n* = 48) stratified by age and daily dose.

Age	Dose	Number of children	AUC_0–24SS_ (mg/L^.^h)		C_max_ (mg/L)	
			Median	[Min–Max]	Median	[Min–Max]
	**<10 mg/kg**	16	78.5	56.63–158.09	7.35	5.49–9.16
**<10 years**	**10 to <20 mg/kg**	16	127.52	59.23–243.23	10.1	6.95–15.8
	**≥20 mg/kg**	5	189.8	139.3–215.9	11.193	9.36–14.1
	**<10 mg/kg**	7	98.9	75.27–321.90	7.306	5.10–21.2
**≥10 years**	**10–20 mg/kg**	4	113.7	102.2–271	11.175	9.87–19.5
	**≥20 mg/kg**	0	-	-	-	-

**Abbreviations:** AUC_0–24ss_, area under the concentration time curve from 0–24 hours at steady state; C_max_, maximum plasma concentration; MDR-TB, multidrug-resistant tuberculosis.

### Simulated exposures

**Fig 2:**
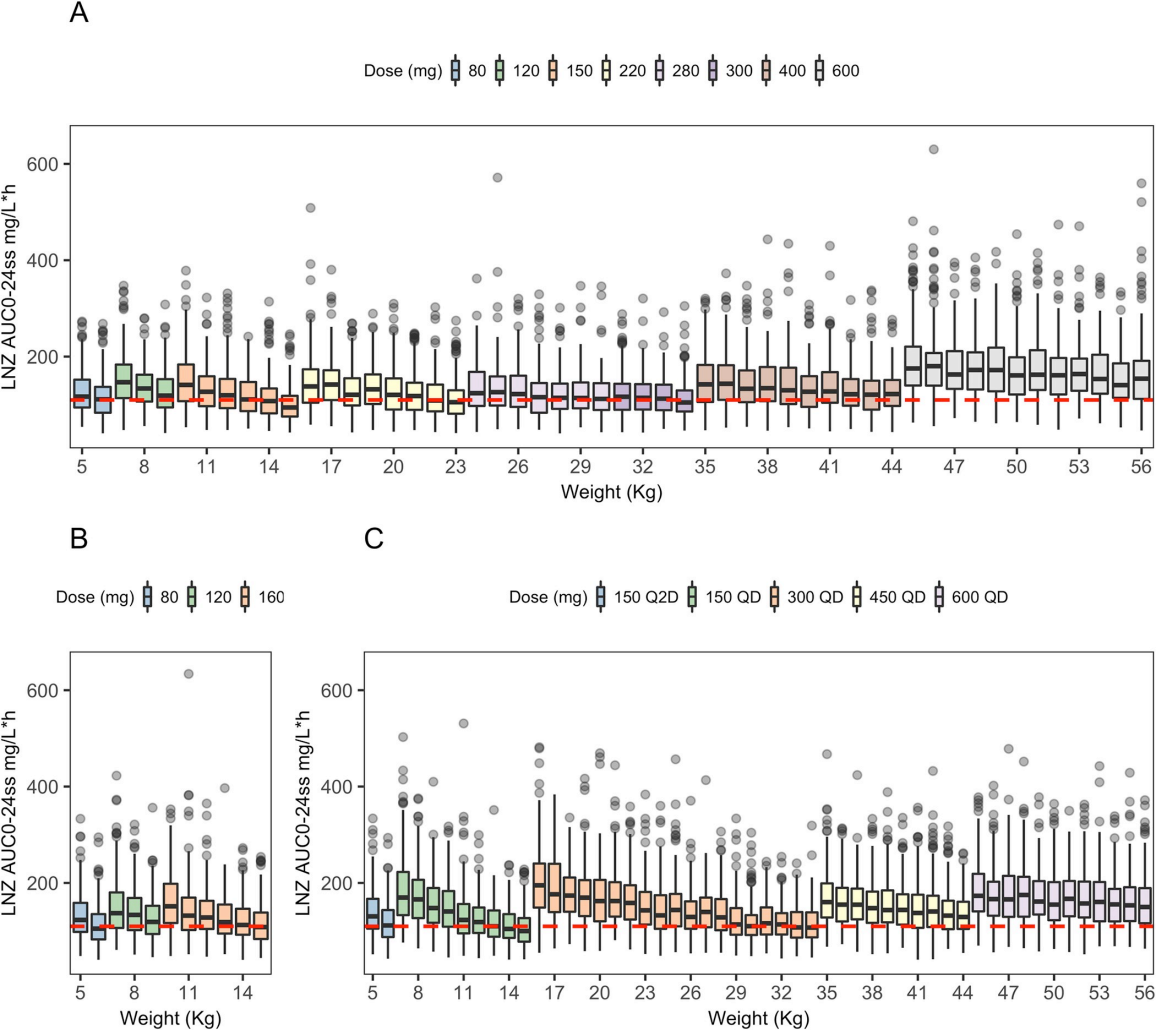
Simulated linezolid exposures in children with model-identified optimal doses. Simulated linezolid AUC_0–24ss_ versus body weight using the linezolid doses listed in Table 4 for (a) model-identified optimal doses independent of formulation, (b) optimal doses for the 20 mg/mL suspension, (c) optimal doses for the 600 mg tablet administered in one-fourth tablet increments; the red hashed line indicates the adult target exposure (AUC_0–24ss_ 110 mg/L^.^h). AUC_0–24ss_, area under the concentration time curve from 0 to 24 hours at steady state; LNZ, linezolid; Q2D, once every other day; QD, once daily.

The proposed weight-banded doses across weights that approximated the emerging AUC targets reported in adults with MDR-TB receiving a 600 mg once-daily dose are shown in Table 4. The expected linezolid exposures (AUC_0–24ss_) across weights from simulations using the final model and this weight-banded dosing strategy are shown in Fig 2.

**Table 4:** Proposed weight-banded once-daily doses of linezolid for children treated for MDR-TB approximating the exposure in adults receiving a 600 mg once-daily dose (AUC_0–24ss_ 110 mg^.^h/L).

	Formulation-independent optimal dosage*		20 mg/mL suspension		600 mg tablet^#^	
Weight band (kg)	Optimal mg dose, once daily	Dose range for weight band (mg/kg)	Optimal mg dose, once daily	Dose range for weight band (mg/kg)	Optimal mg dose, once daily	Dose range for weight band (mg/kg)
5 to <7	80	11.4–16.0	80 (4 mL)	11.4–16.0	150 alt days (1/4 tab)	—
7 to <10	120	12.0–17.1	120 (6 mL)	12.0–17.1	150 (1/4 tab)	15.0–21.4
10 to <16	150	9.4–15.0	160 (8 mL)	10.0–16.0	150 (1/4 tab)	9.4–15.0
16 to <24	220	9.2–13.8	—	—	300 (1/2 tab)	12.5–18.8
24 to <31	280	9.0–11.7	—	—	300 (1/2 tab)	9.7–12.5
31 to <35	300	8.6–9.7	—	—	300 (1/2 tab)	8.6–9.7
35 to <44	400	9.1–11.4	—	—	450 (3/4 tab)	10.2–12.9
>44	600	13.6 -	—	—	600 (1 tab)	13.6 -

*These optimal doses were identified without considering formulation strength.

^#^Possible doses were constrained to 1/4, 1/2, and 3/4 tablet options.

**Abbreviations:** alt, alternate days; AUC_0–24ss_ = area under the concentration time curve from 0–24 hours at steady state; MDR-TB, multidrug-resistant tuberculosis.

### Safety

Seventeen children were included in the safety analysis (median age 4.7 years; range 0.6 to 15.3) and followed for a median duration of 17.7 months on linezolid (IQR 7.5 to 19.7). Ten patients (59%) experienced at least 1 adverse event possibly attributed to linezolid, the most frequent being low haemoglobin, with 12 events in 10 patients (Table 5). There were three grade 3 and two grade 4 events in 5 (23%) participants, all anaemia. The linezolid dose was adjusted after 4 (23.5%) events; linezolid was temporarily interrupted and restarted after 5 (29.4%) events, and 4 (23.5%) participants permanently discontinued linezolid due to adverse events. For the single episode of peripheral neuropathy, which occurred in a 13-year-old female, linezolid was temporarily interrupted, and the patient was treated with gabapentin, resulting in symptomatic improvement. The linezolid was restarted at a lower dose after symptoms resolved with no sequelae, and symptoms did not recur at the lower dose. We did not identify any events of optic neuropathy.

**Table 5:** Adverse events, possibly, probably or definitely attributed to linezolid, in children treated for MDR-TB with linezolid (*n* = 17), by grade.

Adverse event	No. of patients with event	Grade 1	Grade 2	Grade 3	Grade 4	Total no. of events	Event rate (per 100 person-years)
**Peripheral neuropathy**	1	1	0	0	0	1	4.8
**Low haemoglobin**	10	5	2	3	2	12	57.2
**Low platelets**	2	1	1	0	0	2	9.5
**Low white blood cell count**	1	1	0	0	0	1	4.8

Seventeen patients followed for a median time of 17.7 months (IQR: 7.5–19.7 months); total person-years = 21.0.

**Abbreviations:** IQR, interquartile range; MDR-TB, multidrug-resistant tuberculosis.

The median linezolid exposure was associated with occurrence of a grade 3 or 4 linezolid-related adverse event (*p* = 0.020) but not with occurrence of any grade linezolid-related adverse event (*p =* 1.000) (Table 6). The C_min_ and AUC were highly correlated (pairwise correlation coefficient = 0.7737, *p =* 0.0003). The median linezolid C_min_ was not significantly associated with either any grade (*p =* 0.696) or with a grade 3 or 4 (*p =* 0.206) linezolid-related adverse event.

**Table 6:** Comparison of median linezolid AUC_0–24ss_ by occurrence of linezolid-related adverse events in children with MDR-TB (*n* = 17).

	Any grade linezolid-related adverse event			Grade 3 or 4 linezolid-related adverse event		
	Adverse event (*n* = 5)	No adverse event (*n* = 12)	*p*-Value*	Adverse event (*n* = 10)	No Adverse event (n = 7)	*p*-Value*
**Median linezolid AUC**_**0–24ss**_ **(IQR)**	110 (81–229)	105 (83–139)	1.000	229 (121–249)	95 (79–117)	0.020

*Wilcoxon rank sum test

**Abbreviations:** AUC_0–24ss_, area under the concentration time curve from 0–24 hours at steady state; IQR, interquartile range; MDR-TB, multidrug-resistant tuberculosis.

**Fig 3:**
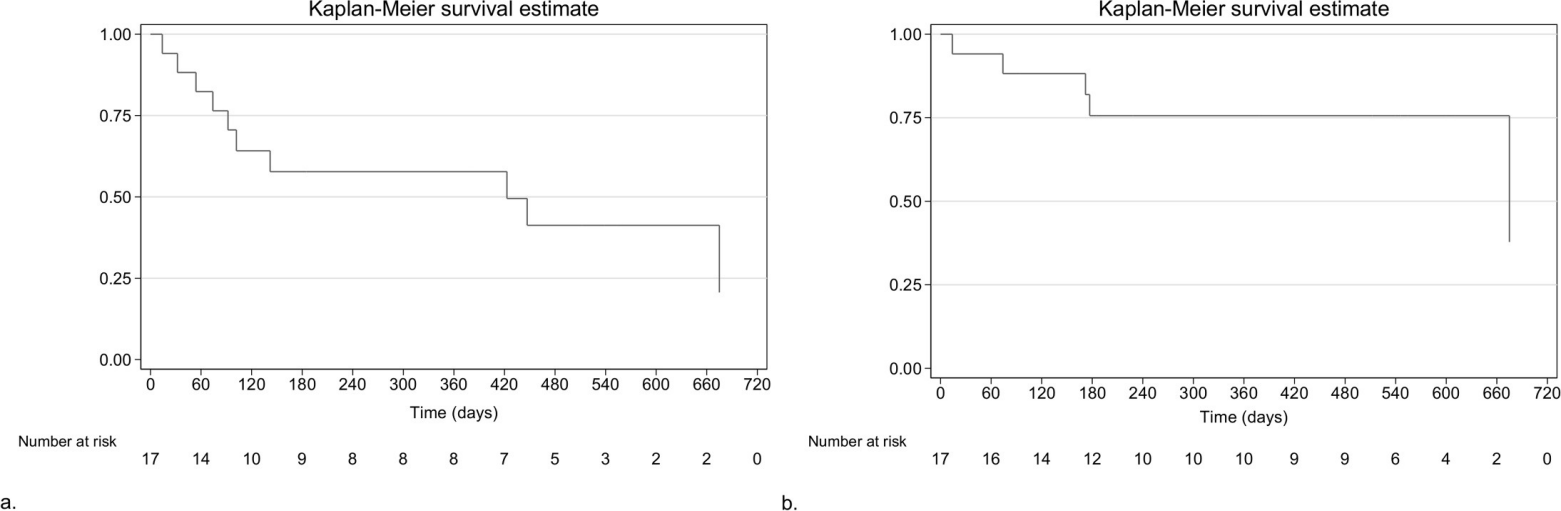
Time to linezolid-related adverse events in children treated for MDR-TB. (a) Kaplan-Meier survival curve for time (in days) to an adverse event of any grade at least possibly related to linezolid; (b) Kaplan-Meier survival curve for time (in days) to a grade 3 or 4 adverse event at least possibly related to linezolid. MDR-TB, multidrug-resistant tuberculosis.

**Fig 4:**
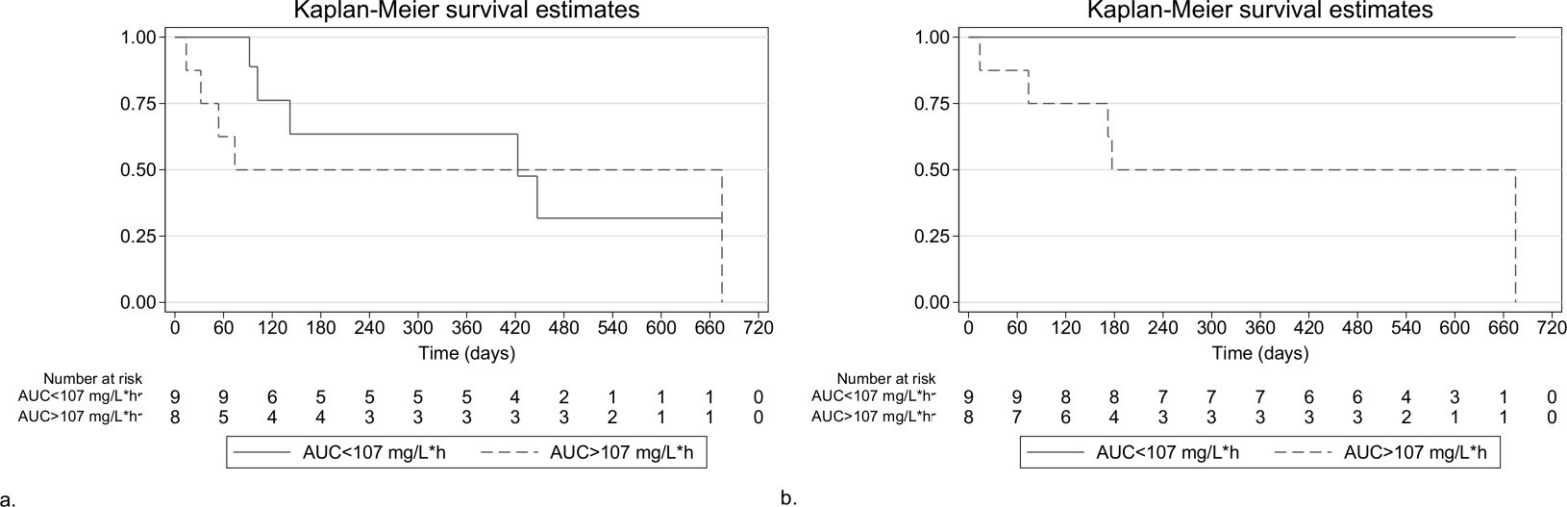
Time to linezolid-related adverse events by linezolid exposures in children treated for MDR-TB. (a) Kaplan-Meier survival curves for time to first linezolid-related adverse event of any grade by linezolid-related AUC_0–24ss_ < 107 mg/L*h versus AUC_0–24ss_ < 107 mg/L*h (log-rank *p* = 0.5895); over a 450-day follow-up, the RMST was not significantly different by AUC_0–24ss_ (295 days if AUC_0–24ss_ < 107 mg/L*h; 222 if AUC_0–24ss_ > 107 mg/L*h [*p =* 0.344]). (b) Kaplan-Meier survival curves for time to grade 3 or 4 linezolid-related adverse event by linezolid-related AUC_0–24ss_ < versus > 107 mg/L*h; log-rank *p* = 0.0121. AUC_0–24ss_ = steady state area under the concentration time curve from 0–24 hours; MDR-TB, multidrug-resistant tuberculosis; RMST, restricted mean survival time.

For children with an adverse event, the median (IQR) time to any linezolid-related adverse event was 3.2 months (1.8 to 13.9) (*n* = 10), and time to grade 3 or 4 linezolid-related adverse event was 2.4 months (1.8 to 4.7) (*n* = 5). The Kaplan-Meier survival curves for time to linezolid-related adverse events are displayed in Fig 3. The time to grade 3 or 4 linezolid-related adverse event differed by linezolid exposure (log-rank *p =* 0.0121), but not the time to any linezolid-related adverse event (log-rank *p =* 0.5895, RMST *p =* 0.344) (Fig 4). Because there was crossing of the survival curves for time to any grade linezolid-related adverse event by linezolid exposure (Fig 4A), we censored the curve at 360 days, but there was still no difference (log-rank *p* = 0.360). Kaplan-Meier survival curves for time to linezolid-related adverse events by C_mins_ are shown in S2 Fig. There were no differences by linezolid C_min_ in time to any grade (log-rank *p =* 0.9267) or grade 3 or 4 (log-rank *p =* 0.5694) linezolid-related adverse event.

## Discussion

This study provides data on linezolid pharmacokinetics in children with TB to inform evidence-based dosing. Linezolid-related adverse events were frequent and occasionally of high grade (grade 3 or 4) in children treated long term for MDR-TB.

The linezolid plasma AUCs seen in our cohort were higher than expected compared to previously published values in children (58 mg/L^.^h for children 3 months to 11 years of age, and 95 mg/L^.^h for 12 to 17 years of age following a 10 mg/kg dose) [19]. The previously published paediatric data reported on single intravenous doses only, so direct comparison to our data is challenging. In certain age groups, exposures in our paediatric cohort were even higher than expected target exposure in adults. These differences could be related to previously unidentified drug-drug interactions with other anti-TB drugs. No association was found between any pharmacokinetic parameters and concomitant drugs in our study; however, the study may have been underpowered to detect these. Linezolid has complex metabolism, and as previously noted, there is substantial variability in linezolid pharmacokinetics between individuals and by disease state [17, 18, 33, 34]. Differences in bioavailability, including formulation effects, should also be considered. The formulations used in our study were all nongeneric, as used in most adult studies to date, and no differences were observed in pharmacokinetic parameters by formulation administration (whole versus crushed versus suspension) in our cohort.

Our study combined linezolid pharmacokinetic data after both single and multiple doses. Usually, pharmacokinetic parameters (CL, V of distribution, and absorption rate constant) derived based on single-dose pharmacokinetic data can predict well multiple-dose pharmacokinetics, if the pharmacokinetics are linear and there are no time-dependent trends, such as autoinduction or nonlinear elimination. This was the case for linezolid. The multiple-dose data confirmed the absence of these trends in this paediatric population, and the single-dose data were valuable in informing the relationship between children’s CL and weight and/or age, which forms the basis for establishing the dosing algorithm.

Additional data on linezolid pharmacokinetics in children treated for MDR-TB are important to confirm all of these findings.

At the linezolid doses used in this study, 10 of 17 children treated long term experienced a linezolid-related adverse event, all of whom had anaemia in addition to some other, less frequent events. This is similar to adult data and to a previous summary of published case series and case reports in which 9 of 18 children had a linezolid-related event [9]. In our study, regular monitoring was able to identify anaemia at mild grades in most children. The approach of temporarily interrupting the linezolid until the haemoglobin had improved, followed by re-introduction at a lower dose (usually half the previous dose), was generally well tolerated. Five children experienced a grade 3 or 4 anaemia, 4 of whom permanently discontinued linezolid. Although all patients recovered from their anaemia without sequelae, the frequency and severity of these events highlights the importance of careful monitoring of haematological parameters during long-term linezolid treatment. In our study, once haemoglobin values began to drop, they often fell rapidly, so the first signs of a falling haemoglobin should prompt more frequent testing, and there should be a low threshold for temporarily interrupting doses, depending on the importance of linezolid to the efficacy of the regimen. The cumulative incidence of adverse events increased with longer duration of treatment; however, there were events detected even in the first 60 days, including grade 3 and 4 events. Reducing the duration of linezolid treatment would likely reduce, but not eliminate, the risk of adverse events, and careful safety monitoring will be necessary in future clinical trials and in routine care, regardless of the duration of linezolid therapy. Reducing the linezolid dose would be expected to reduce its activity and may increase the risk of acquired resistance. The risk to children, who tend to have paucibacillary disease, would likely be low, especially when the child has already been on treatment for weeks or more with effective companion drugs in the regimen, and the bacillary burden would have been substantially reduced. However, there is still uncertainty about the risk of acquired resistance and the efficacy of these lower doses of linezolid, and an improved understanding of how to weigh up this risk versus benefit is an area for future research.

Grade 3 or 4 events were strongly associated with higher linezolid exposures. The lower doses we have proposed may reduce this risk. Therapeutic drug monitoring may also be a consideration to explore further to reduce the risk of these more severe events.

Peripheral neuropathy was detected in only one child, a 13-year-old female, and improved with temporary discontinuation of linezolid and gabapentin treatment, and it ultimately resolved without sequelae after recommencing a reduced linezolid dose. To our knowledge, there is no reason to believe that children do not develop linezolid-related peripheral neuropathy, although the risk may be lower than in adults. Peripheral neuropathy presents with symptoms of pain, numbness, and tingling in a stocking-glove distribution, and signs of weakness, reduced deep-tendon reflexes, and loss of sensation of vibration, proprioception, and temperature. All of these signs and symptoms can be challenging to elicit in young children. To our knowledge, there are no validated clinical approaches for screening for peripheral neuropathy that can be used in young children. The gold standard for diagnosis is nerve conduction studies, which are invasive and specialised tests not widely available in high–TB-burden settings. The implication is that peripheral neuropathy may be difficult to identify in young children, and thus there is a risk of underdiagnosis. This is an important consideration when weighing the risk to benefit for the use of linezolid in children with MDR-TB. We did not identify any other serious, more rare events such as optic neuropathy or lactic acidosis. This is reassuring but should be interpreted cautiously, as the number of children included here is modest, and formal ophthalmologic examinations were not done. Lactate levels and pancreatic enzymes were not monitored, which is also a limitation. However, in no children were clinical symptoms or signs suggestive of optic neuropathy, lactic acidosis, or pancreatitis reported or suspected.

Using the final model, our simulations identified weight-banded doses that would achieve the proposed target exposure, based on emerging data in adults with MDR-TB receiving a 600 mg daily dose (Table 4, Fig 2). Doses used for children in routine care will be constrained by available formulations. A 20 mg/mL suspension exists but is not widely available due to cost, variable supply, or lack of in-country registrations, and most settings only have access to the unscored 600 mg tablet. Pragmatic doses using the available formulations result in less optimal exposures in some weight bands, which may increase the risk of adverse effects (Table 4, Fig 2). This should not limit the use of linezolid in children when indicated; however, more flexible, child-friendly formulations are urgently needed.

These new proposed doses are lower than those used in this study and are currently used in routine care for MDR-TB in most settings. Lower doses in children would likely reduce the risk of adverse events, which were frequent in this cohort, although this would need to be confirmed in further studies. Because exposures with these doses in children would be expected to approximate those in adults after a 600 mg daily dose, the efficacy would be expected to be as good or better in most children compared to adults receiving this dose, as children often have paucibacillary TB. Careful evaluation of the pharmacokinetics, safety, and treatment outcomes in children treated with these proposed new, weight-banded doses of linezolid is needed.

The linezolid exposure targeted clearly has an impact on paediatric dosing studies and recommendations. The target exposure chosen here (110 mg/L^.^h) is based on robust data from 2 adult TB trials. This target was chosen because it was taken from recent studies that also included efficacy and toxicity data. The optimal linezolid target exposure and the optimal dosing strategy for linezolid has not yet been established in adults. An ongoing phase III trial (ZeNix, NCT03086486) is evaluating the efficacy and safety of multiple doses and durations of linezolid in combination with bedaquiline and pretomanid in adults with MDR-TB. ZeNix will provide important data to inform future linezolid dosing recommendations in adults. A free AUC/minimum inhibitory concentration (MIC) ratio of >100 has been proposed for efficacy based on hollow fibre model data [35, 36]. A linezolid free-AUC/MIC ratio of <96 or a trough <2 mg/L have been proposed as targets to reduce the risk of adverse effects [31, 35, 36]. However, more data are needed to understand how clinically relevant these targets are in adults and in children of different ages. We are planning additional work to better characterise the relationship between linezolid pharmacokinetics and adverse events using current and future data and pharmacokinetic-pharmacodynamic modelling. Due to its excellent penetration into CSF, linezolid may be an important component of treatment regimens for TB meningitis. Higher doses of linezolid than used for other forms of TB may be needed in order to achieve target exposures in CSF. This is an important area for future research.

There is a growing awareness of the importance of drug exposure at the site of disease, which is influenced by penetration of drugs into different areas of TB lesions [37]. Targeting plasma AUC/MIC does not take this key aspect into consideration. Targeting drug exposures that are linked with efficacy in TB patients with the spectrum of TB disease and lung pathology does incorporate this aspect and, in our estimation, is a more appropriate target given the current state of knowledge. Regardless, paediatric linezolid dosing recommendations may need to evolve as additional data on optimal dosing in adults become available.

The study has a number of important limitations. Although this is a relatively large study of intensively sampled pharmacokinetics in children, the numbers of included children remain small, especially regarding potentially relevant subgroups. There were few very young and small children included in this analysis, and none under 6 months of age. There are data in neonates and young infants indicating that the CL of linezolid is associated with postnatal age, with children <8 days of age having lower CL than those 8 days to 12 weeks of age (i.e., CL rapidly increased in the first week of life) [38]. Our proposed doses for smaller children should be interpreted with caution because infants younger than 6 months were not included. For clinicians in the field responsible for caring for the occasional young, small child with MDR-TB, some practical guidance on dosing—even with inherent limitations—is, however, still valuable. Additionally, there were few children with HIV infection or undernutrition. We are unaware of clinically significant drug-drug interactions between antiretrovirals and linezolid or of other previously described effects of HIV or undernutrition on linezolid pharmacokinetics. However, these are important covariates to consider, and our small data set is underpowered to detect effects of HIV infection or undernutrition that could be clinically significant. A larger study, or pooling of data, would help better characterise the effect of any of these important covariates. Additionally, we did not detect an effect of formulation (whole tablets versus crushed tablets versus suspension). The small sample size and confounding by age (young children received suspension and older children whole tablets) limited our ability to detect such formulation effects if they existed.

There were also limitations to our safety analysis. The modest number of children receiving long-term linezolid and contributing to safety data limits the precision of our estimates of the proportion of children with adverse events detected. The fact that more rare events, such as optic neuropathy, were not detected does not mean they might not be observed with larger numbers of patients. HIV-infected patients receiving linezolid may be at a higher risk of adverse effects because of overlapping mitochondrial toxicity with some antiretroviral drugs, especially the non-nucleoside reverse transcriptase inhibitors. This remains an important area for future research.

## Conclusion

This study provides urgently needed data on linezolid pharmacokinetics and prospective safety data in children with TB. Linezolid exposures were higher than expected, and adverse events were common and occasionally severe. Based on these data, we have proposed, for the first time, paediatric weight-banded doses approximating a 600-mg–daily adult linezolid dose. Clear guidance on the optimal dose of linezolid for children with TB is urgently needed given its growing importance in MDR-TB treatment regimens. These data have informed revised WHO-recommended dosing in children.

## Supporting information

S1 STROBE Checklist Completed STROBE checklist. STROBE, Strengthening the Reporting of Observational Studies in Epidemiology.
(DOCX)

S1 Text MDRPK2 protocol Statistical Considerations section (February 11, 2015). (PDF)

S1 Table Summary of low platelets, low white blood cell count, and peripheral neuropathy adverse event grading from the DAIDS Adverse Event Grading Tables versions 1.0, 2.0, and 2.1. DAIDS, Division of AIDS.
(DOCX)

S2 Table Summary of low haemoglobin adverse event grading from the DAIDS Adverse Event Grading Tables versions 1.0, 2.0, and 2.1. DAIDS, Division of AIDS.
(DOCX)

S3 Table Coefficients and confidence intervals (95%) of several covariate models tested during the pharmacokinetic analysis. (DOCX)

S4 Table Pharmacokinetic parameters used to derive the target AUC for linezolid in adults after 600 mg once daily. AUC, area under the concentration time curve.
(DOCX)

S1 Fig PK profiles of participants contributing on more than one occasion to the pharmacokinetic analysis. PK, pharmacokinetic.
(DOCX)

S2 Fig (a) Kaplan-Meier survival curve for time (in days) to an adverse event of any grade at least possibly related to linezolid by C_min_ < or > 2 mg/L. (b) Kaplan-Meier survival curve for time (in days) to a grade 3 or 4 adverse event at least possibly related to linezolid by C_min_ < or > 2 mg/L. C_min_, minimum linezolid plasma concentration.
(DOCX)

S1 Data Linezolid PK data set. PK, pharmacokinetic.
(CSV)

S2 Data Linezolid PK data set data dictionary. PK, pharmacokinetic
(XLSX)

S3 Data Linezolid demographics and safety data. (XLSX)

## Abbreviations

AUC_0–24ss_: area under the concentration time curve from 0 to 24 hours at steady state
BSV: between-subject variability
CL: clearance
C_min_: minimum linezolid concentration
CSF: cerebrospinal fluid
DAIDS: Division of AIDS
FOCE: first order conditional estimation
HAZ: height-for-age z-score
IQR: interquartile range
K_a_: rate of absorption
LC MS/MS: liquid chromatography tandem mass spectrometry
MDR-TB: multidrug-resistant tuberculosis
MIC: minimum inhibitory concentration
QC: quality control
RMST: restricted mean survival time
RSE: relative standard error
SCM: stepwise covariate modelling
STROBE: Strengthening the Reporting of Observational Studies in Epidemiology
V: volume
VPC: visual predictive check
WAZ: weight-for-age z-score
WHO: World Health Organisation
XDR-TB: extensively drug-resistant tuberculosis
