# Supplementary material for: Pharmacokinetics, optimal dosing, and safety of linezolid in children with multidrug-resistant tuberculosis: Combined data from two prospective observational studies
Source: PLoS Med. 2019 Apr 30;16(4):e1002789. doi: 10.1371/journal.pmed.1002789 (PMC6490911; doi:10.1371/journal.pmed.1002789)
Supplement: S1 Text — (PDF) [file pmed.1002789.s002.pdf]

7. STATISTICAL CONSIDERATIONS

Sample size estimations

PK of L, M, and Lzd: Repeated simulation and re-estimation ("sse") of clinical trials were performed to evaluate our proposed design in terms of its ability to identify: a) the Population PK (PopPK)parameters and between-subject variability (BSV) with sufficient precision, b) the maturation component of drug clearance (if present) in children <2 y, and c) the effect of tablet crushing on the bioavailibility of L and M. Uncertainty in parameter estimates (RSE) of <10% was deemed acceptable for the PopPK parameters and <20% for the maturation parameter. Our simulations indicated that for the maturation parameter it is important that multiple sampling occasions are implemented, and that a large fraction of young patients should be enrolled in the study, since otherwise the uncertainty in the maturation parameter increases considerably. A design in which only one PK curve was taken at study start was not able to identify the maturation, and led to high uncertainty in parameter estimates. The design that was able to capture the maturation function with adequate certainty while limiting the costs, was the design with *a minimum* of 80 children enrolled, an enriched age distribution (60% of children < 2yrs), a sampling schedule with a limited PK curve at start (t = 1, 4, 10 hours) and additional pre-dose samples after 12 and 18 months of treatment in some individuals.

Treatment response: L - Assuming that close to 80% of culture confirmed cases will convert at 2 months, a two group t-test with a 0.05 two-sided significance level will have 80% power to detect the difference between patients who convert at 2 months with a mean L $AUC_{(0-\infty)}$, μ_1, of 27.0 µg*h/mL vs. those who fail to convert at 2 months with a mean L $AUC_{(0-\infty)}$, μ_2, of 37.0 µg*h/mL, assuming a common standard deviation (SD) of 8.5, when the sample sizes in the two groups are 25 and 8, respectively (a total sample size of 32). *M* - Assuming that close to 80% of culture confirmed cases will convert at 2 months, a two group t-test with a 0.05 two-sided significance level will have 80% power to detect the difference between patients who convert at 2 months with a mean M $AUC_{(0-\infty)}$, μ_1, of 18.0 µg*h/mL vs. failure to convert with a mean M $AUC_{(0-\infty)}$, μ_2, of 28.0 µg*h/mL, a difference in means of -10.0 µg*h/mL, assuming a common SD of 4.277, when the sample sizes in the two groups are 8 and 3, respectively (a total sample size of 10).

Bioavailability: Relative bioavailibility (F_{rel}) of the crushed tablet was estimated as a model parameter in the Population PK model. The simulations used an F_{rel} of 80%, considered the upper limit of clinical relevance. An uncertainty of <5% in F_{rel} was assumed to be sufficiently precise and used as the goal in simulations. The design identified from our simulations assumed at least 10 patients >5y were enrolled in the cohort to study the effect of tablet crushing on relative bioavailability, and a full PK profile was taken on two occasions (cross-over design). The chosen design requires 14 samples per patient in this cohort (7 at each occasion), and estimated the relative bioavailability with sufficient precision.

PopPK modeling methods

The PopPK model developed in this study will capture the often-complex dynamics of PK in children. We will attempt to apply allometric scaling in our model, a proven approach to capture differences in PK between children of differing body sizes and shapes. However, some metabolic changes are not well captured by allometric scaling alone. While weight is often a notable predictor of clearance (CL) and strongly correlated with a child's age, age may be an additional predictor of PK independent of weight, due to the maturation of enzymes in the liver or kidney function during development, as is case for L.[42] We will attempt to capture any longitudinal changes in clearance and other PK parameters using a maturation function. Model building will be guided by both numeric and visual diagnostics. We will apply state-of-the-art model evaluation diagnostics, such as the visual predictive

check, npde, and bootstrap. As various dose levels will be studied, diagnostics will be either stratified or prediction-corrected, if required. For evaluation of covariates, we will apply stepwise covariate model building. *Interim analyses:* Given the limited data on M PK in young children, we will perform an interim analysis when the first 15 children <8y have completed M PK, evaluating PK and available safety data. Interim analysis of Lzd PK and safety will also follow after completion of n=15 participants. We will also do formal interim analysis of PK and safety of M, L, and Lzd when 50% of the sample has completed (n=50). Using this data we will assess the target attainment at the optimized doses as previously described, and add this data into the population PK models to further refine our optimized dosing. Based on these interim analyses, we may alter our dosing taking into consideration any safety concerns. For each drug, our major goal is to ensure that target exposures from adult population (see C3d, C3e, C3f) have been reached in every child irrespective of weight and age.

Other analysis

Treatment response: PK-PD modelling: Using modeling, we will evaluate whether the applied regimens are capable of achieving the prior defined MIC or AUC/MIC. Additionally, we will implement a joint model that will link PK in children to a time-to-event (survival) model, in which the effect of individual drug exposure/C_{max} relative to the MIC as well as other patient characteristics will be correlated to the time to 1st and stable negative culture in liquid medium. The predictor variable AUC and all covariates of interest, including age, HIV status, nutritional status, disease severity, will be assessed to determine for association with any treatment response outcomes. We will also evaluate one and 2 month culture conversion and final treatment outcome.

AEs: Safety will be reported using the cumulative incidence of all and drug-related Grade 3/4 AEs for each drug separately. Any AE grade ≥3 that is at least possibly associated with L, M or Lnz, will trigger review of data by the study team and will result in dose adjustment as clinically indicated. For cardiotoxicity, we will report mean longest QTc, proportion of children with prolonged QTc, mean largest QTc change from pre-dose, and proportion with QTc change from pre-dose >30 ms. A joint PK-PD model will evaluate the relationships between drug exposure and the incidence of safety events, i.e. the cumulative incidence of drug-related grade 3/4 adverse effects (AEs), corrected QT interval (QTc) and change in QTc from pre-dose (L, M).

Bioavailability: Relative bioavailibility (F_{rel}) for crushed tablets will be estimated in the PopPK model on the basis of the data from the bioavailibility cross-over cohort of 10 patients (per drug). The estimate of relative bioavailibility will be based on data collected in children >5 years who are able to swallow the whole tablet. Addition of this data into the model is expected to improve estimation of all model parameters, specifically maturation function. Knowledge gained on potential loss/gain in relative bioavailability following tablet crushing will be built in our final recommended dosing algorithm for younger children who are not able to swalllow the whole tablet, in order to account for expected changes in drug exposures due to altered relative bioavailibiltiy.

Acceptability: We will report summary statistics for each acceptability measure, by drug and age group (<5y, >5y). Using logistic regression, we will evaluate covariates (age, formulation, HIV status) associated with drug acceptability. Acceptability will be analyzed separately for children <5 yrs and for children >5 yrs. For children participating in the crossover design bioavailability assessment, we will report a within person comparison of acceptability of crushed vs whole formulation.
