# Supplementary material for: Pharmacokinetics, optimal dosing, and safety of linezolid in children with multidrug-resistant tuberculosis: Combined data from two prospective observational studies
Source: PLoS Med. 2019 Apr 30;16(4):e1002789. doi: 10.1371/journal.pmed.1002789 (PMC6490911; doi:10.1371/journal.pmed.1002789)
Supplement: S1 Table — DAIDS, Division of AIDS. (DOCX) [file pmed.1002789.s003.docx]

| **S1 Table. Summary of *low platelets, low white blood cell count, and peripheral neuropathy* adverse event grading from the Division of AIDS Adverse Event Grading Tables Versions 1.0, 2.0 and 2.1.** | | | | |
| --- | --- | --- | --- | --- |
|  | **Grade 1** | **Grade 2** | **Grade 3** | **Grade 4** |
| **Low platelets (cells/mm3; *cells/L*)** | 100,000 to <125,000 | 50,000 to <100,000 | 25,000 to <50,000 | < 25,000 |
| **Low white blood cell count (<7 days of age) (cells/mm3; cells/L)** | 2,000 to 2,499  2.000 x10^9^ to 2.499 x 10^9^ | 1,500 to 1,999  1.500 x 10^9^ to 1.999 x 10^9^ | 1,000 to 1,499  1.000 x 10^9^ to 0.499 x 10^9^ | < 1,000  < 1.000 x 10^9^ |
| **Peripheral neuropathy (Neurosensory alteration)** | Minimal paresthesia causing no or minimal interference with usual social & functional activities OR No symptoms with sensory alteration on examination | Sensory alteration or paresthesia causing greater than minimal interference with usual social & functional activities | Sensory alteration or paresthesia causing inability to perform usual social & functional activities | Disabling sensory alteration or paresthesia causing inability to perform basic care functions |
