# Supplementary material for: Pharmacokinetics, optimal dosing, and safety of linezolid in children with multidrug-resistant tuberculosis: Combined data from two prospective observational studies
Source: PLoS Med. 2019 Apr 30;16(4):e1002789. doi: 10.1371/journal.pmed.1002789 (PMC6490911; doi:10.1371/journal.pmed.1002789)
Supplement: S2 Table — DAIDS, Division of AIDS. (DOCX) [file pmed.1002789.s004.docx]

| **S2 Table. Summary of *low haemoglobin* adverse event grading from the Division of AIDS Adverse Event Grading Tables Versions 1.0, 2.0 and 2.1.** | | | | |
| --- | --- | --- | --- | --- |
|  | **Grade 1** | **Grade 2** | **Grade 3** | **Grade 4** |
| **DAIDS Table for Grading the Severity of Adult and Pediatric Adverse Events version 1.0, December 2004, updated August 2009** | | | | |
| **Adult and Pediatric**  **≥ 57 days**  **(HIV POSITIVE ONLY)** | 8.5 – 10.0 g/dL  *5.24 – 6.23 mmol/L* | 7.5 – 8.4 g/dL  *4.62–5.23 mmol/L* | 6.50 – 7.4 g/dL  *4.03–4.61 mmol/L* | < 6.5 g/dL  *< 4.03 mmol/L* |
| **Adult and Pediatric**  **≥ 57 days**  **(HIV NEGATIVE ONLY)** | 10.0 – 10.9 g/dL  *6.18* – 6.79 *mmol/L*  OR  Any decrease 2.5 – 3.4 g/dL  *1.58 – 2.13 mmol/L* | 9.0 – 9.9 g/dL  *5.55 - 6.17 mmol/L*  OR  Any decrease 3.5 – 4.4 g/dL  *2.14 – 2.78 mmol/L* | 7.0 – 8.9 g/dL  *4.34 - 5.54 mmol/L*  OR  Any decrease  ≥ 4.5 g/dL  *> 2.79 mmol/L* | < 7.0 g/dL  *< 4.34 mmol/L* |
| **DAIDS Table for Grading the Severity of Adult and Pediatric Adverse Events (Version 2.0, November 2014, and Corrected version 2.1, July 2017)** | | | | |
| **≥13 years of age (male only)** | 10.0 to 10.9 g/dL  *6.19 to 6.76 mmol/L* | 9.0 to < 10.0 g/dL  *5.57 to < 6.19 mmol/L* | 7.0 to < 9.0 g/dL  *4.34 to < 5.57 mmol/L* | < 7.0 g/dL  *< 4.34 mmol/L* |
| **≥ 13 years of age (female only)** | 9.5 to 10.4 g/dL  *5.88 to 6.48 mmol/L* | 8.5 to < 9.5 g/dL  5.25 to < 5.88 *mmol/L* | 6.5 to < 8.5 g/dL  *4.03 to < 5.25 mmol/L* | < 6.5 g/dL  *< 4.03 mmol/L* |
| **57 days of age to < 13 years of age**  **(male and female)** | 9.5 to 10.4 g/dL  *5.88 to 6.48 mmol/L* | 8.5 to < 9.5 g/dL  *5.25 to < 5.88 mmol/L* | 6.5 to < 8.5 g/dL  *4.03 to < 5.25 mmol/L* | < 6.5 g/dL  *< 4.03 mmol/L* |
