# Supplementary material for: Pharmacokinetics, optimal dosing, and safety of linezolid in children with multidrug-resistant tuberculosis: Combined data from two prospective observational studies
Source: PLoS Med. 2019 Apr 30;16(4):e1002789. doi: 10.1371/journal.pmed.1002789 (PMC6490911; doi:10.1371/journal.pmed.1002789)
Supplement: S3 Table — (DOCX) [file pmed.1002789.s005.docx]

**S3 Table. Coefficients and Interval Confidents (95%) of several covariate models tested during the pharmacokinetic analysis.**

|  | Covariate-Parameter Model | Coefficient (PE) | SE | | IC_95%_ |
| --- | --- | --- | --- | --- | --- |
| Age-CL | CLAGE = ( 1 + PE*(AGE - 4.58))  CL=CLAGE*TVCL*(WT/70)**0.75 | -0.0141 | 0.0153 | | [-0.044, 0.016] |
| Gender-CL | CLGENDER*_female_* = 1  CLGENDER*_male_* = 1 + PE  CL=CLGENDER*TVCL*(WT/70)**0.75 | -0.0756 | 0.117 | | [-0.305, 0.154] |
| HIV-CL | CLHIV*_0_* = 1  CLHIV*_1_* = 1 + PE  CL=CLHIV*TVCL*(WT/70)**0.75 | 0.0812 | 0.24 | | [-0.389, 0.552] |
| Height-CL | CLHT = ( 1 + PE*(HT - 100.4))  CL=CLHT*TVCL*(WT/70)**0.75 | -0.00181 | 0.0029 | | [-0.007, 0.004] |
| Ethnicity-CL | CLETH*_black_* = 1  CLETH*_mixed_*= 1 + PE  CL=CLETH*TVCL*(WT/70)**0.75 | -0.0427 | 0.12 | | [-0.278, 0.193] |
| WAZ-CL | CLWAZ = ( 1 + PE*(WAZ – 1.06))  CL=CLWAZ*TVCL*(WT/70)**0.75 | 0.0655 | 0.0492 | | [-0.161, 0.031] |
| Age-V | VAGE = ( 1 + PE*(AGE - 4.58))  V=VAGE*TVV*(WT/70) | 0.0111 | | 0.0134 | [-0.037, 0.015] |
| Gender-V | VGENDER*_female_* = 1  VGENDER*_male_* = 1 + PE  V=VGENDER*TVV*(WT/70) | 0.25 | | 0.135 | [-0.015, 0.515] |
| HIV-V | VHIV*_0_* = 1  VHIV*_1_* = 1 + PE  V=VHIV*TVV*(WT/70) | -0.324 | | 0.0566 | [-0.435, -0.213] |
| Height-V | VHT = ( 1 + PE*(HT - 100.4))  V=VHT*TVV*(WT/70) | 0.0021 | | 0.002 | [-0.006, 0.002] |
| Ethnicity-V | VETH*_black_* = 1  VETH*_mixed_*= 1 + PE  V=VETH*TVV*(WT/70) | 0.0528 | | 0.123 | [-0.188,  0.294] |
| WAZ-V | VWAZ = ( 1 + PE*(WAZ – 1.06))  V=VWAZ*TVV*(WT/70) | -0.0054 | | 0.0503 | [-0.104, 0.093] |

Table footnotes: IC: Interval Confident. PE: Parameter estimate. CL: clearance. V: Volume of distribution. TVCL and TVV: Typical value of clearance and volume parameter, respectively. WT: Weight.
