# Supplementary material for: Pharmacokinetics, optimal dosing, and safety of linezolid in children with multidrug-resistant tuberculosis: Combined data from two prospective observational studies
Source: PLoS Med. 2019 Apr 30;16(4):e1002789. doi: 10.1371/journal.pmed.1002789 (PMC6490911; doi:10.1371/journal.pmed.1002789)
Supplement: S4 Table — AUC, area under the concentration time curve. (DOCX) [file pmed.1002789.s006.docx]

| **Parameter** | **Estimate** |
| --- | --- |
| K_a_ (h^-1^) | 2.91 |
| CL_int_ (L/h) | 5.94 |
| V (L) | 50.7 |
| KM (mg/L) | 26 |
| CL_linear_ (L/h)=(1+CL_ratio_)*CRCL | CL_ratio_ =-0.891 |
| BSV_CLint_ (%) | 34 |
| BSV_V_ (%) | 27 |

**S4 Table. Pharmacokinetic parameters used to derive the target area-under the concentration time curve (AUC) for linezolid in adults after 600 mg once daily.**

*Bioavailability was assumed to be 100%. K_a_ = absorption rate constant; CL_int_ = intrinsic clearance; V = volume of distribution; KM= Michaelis-Menten constant (VMAX=CL_int_ x KM); CL_linear_= linear clearance, CRCL=creatinine clearance; BSV = between subject variability.
