# Supplementary material for: Pharmacokinetics, optimal dosing, and safety of linezolid in children with multidrug-resistant tuberculosis: Combined data from two prospective observational studies
Source: PLoS Med. 2019 Apr 30;16(4):e1002789. doi: 10.1371/journal.pmed.1002789 (PMC6490911; doi:10.1371/journal.pmed.1002789)
Supplement: S1 Fig — PK, pharmacokinetic. (DOCX) [file pmed.1002789.s007.docx]

**S1 Fig. Pharmacokinetic (PK) profiles of participants contributing in more than one occasion to the pharmacokinetic analysis.** The PK sample in second occasion of ID 217 was collected after the administration of a crushed tablet formulation.
