# Supplementary material for: Pharmacokinetics, optimal dosing, and safety of linezolid in children with multidrug-resistant tuberculosis: Combined data from two prospective observational studies
Source: PLoS Med. 2019 Apr 30;16(4):e1002789. doi: 10.1371/journal.pmed.1002789 (PMC6490911; doi:10.1371/journal.pmed.1002789)
Supplement: S2 Fig — (a) Kaplan-Meier survival curve for time (in days) to an adverse event of any grade at least possibly related to linezolid by Cmin < or > 2 mg/L. (b) Kaplan-Meier survival curve for time (in days) to a grade 3 or 4 adverse event at least possibly related to linezolid by Cmin < or > 2 mg/L. Cmin, minimum linezolid plasma concentration. (DOCX) [file pmed.1002789.s008.docx]

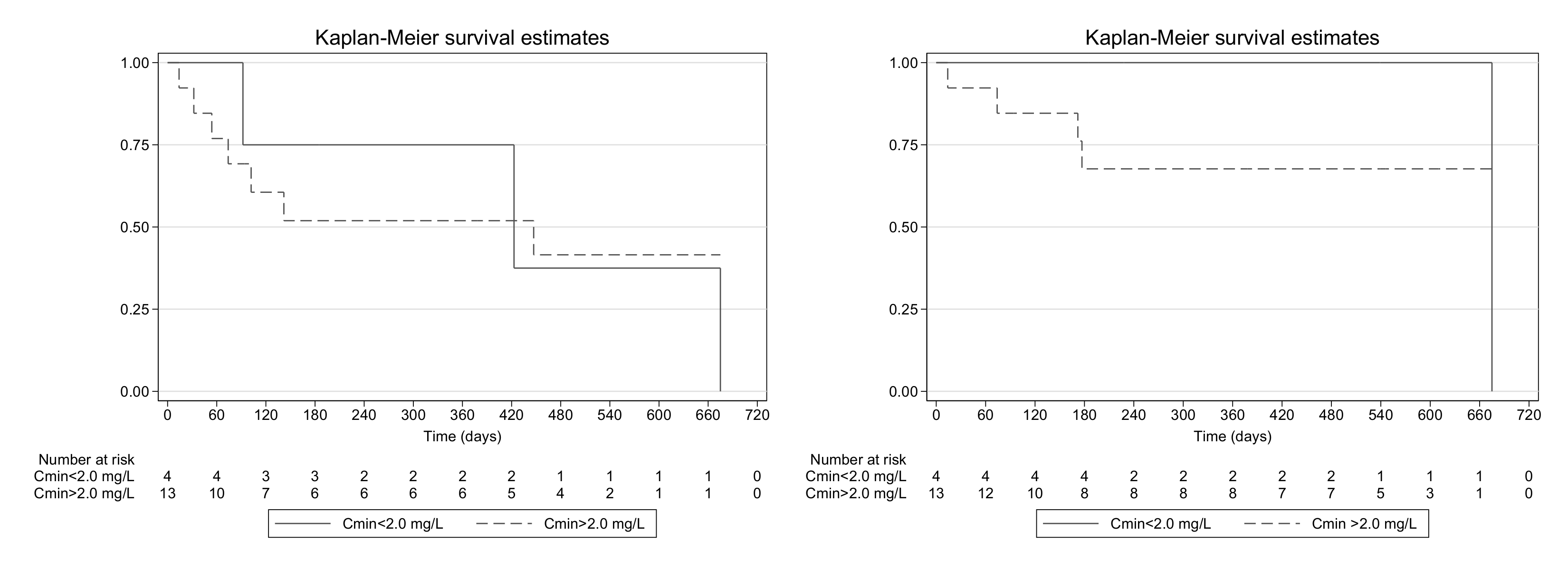


**S2_Fig.** **a) Kaplan-Meier survival curve for time (in days) to an adverse event of any grade at least possibly related to linezolid by minimum linezolid plasma concentration (C_min_) < or > 2 mg/L; b) Kaplan-Meier survival curve for time (in days) to a grade 3 or 4 adverse event at least possibly related to linezolid by C_min_ < or > 2 mg/L.**
